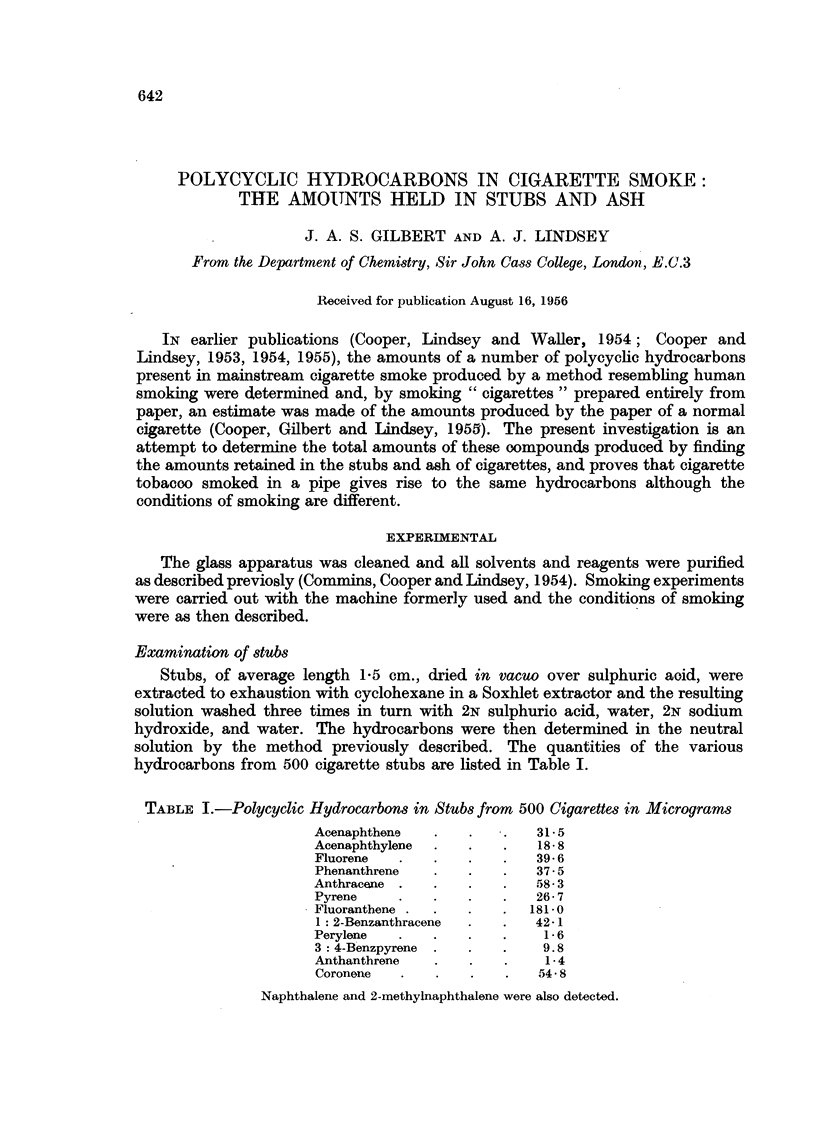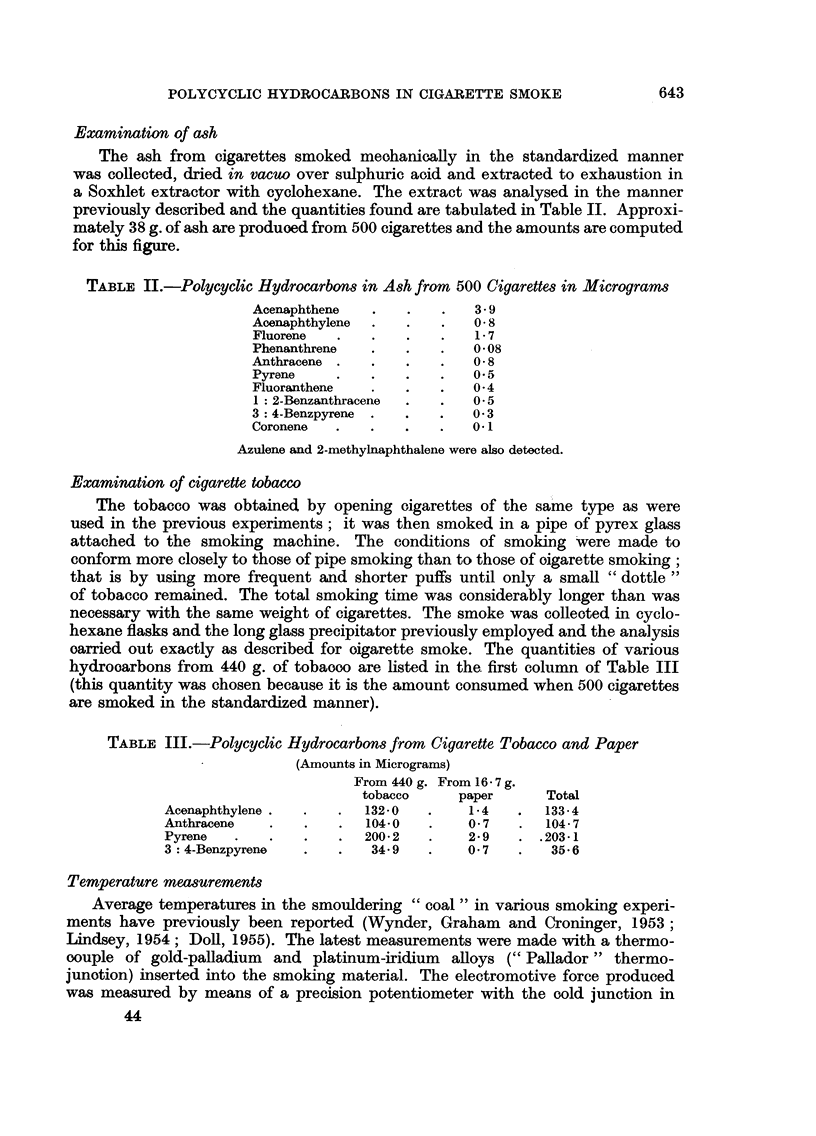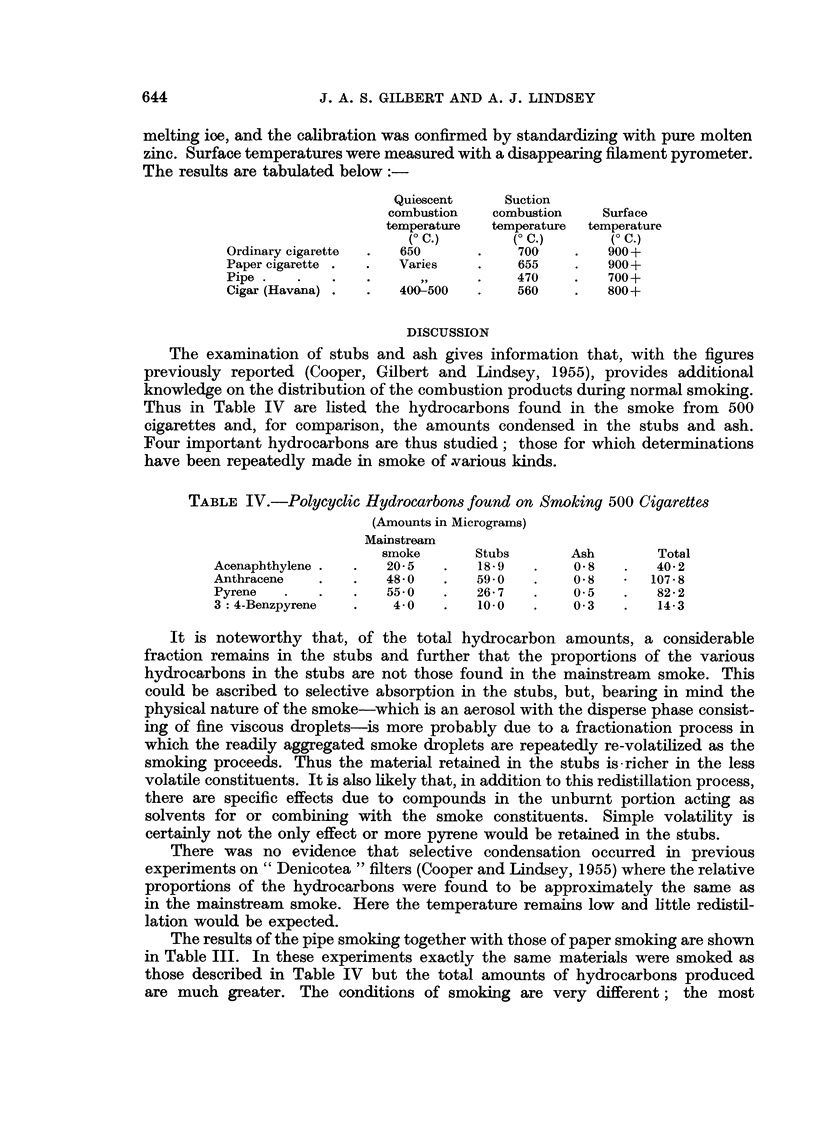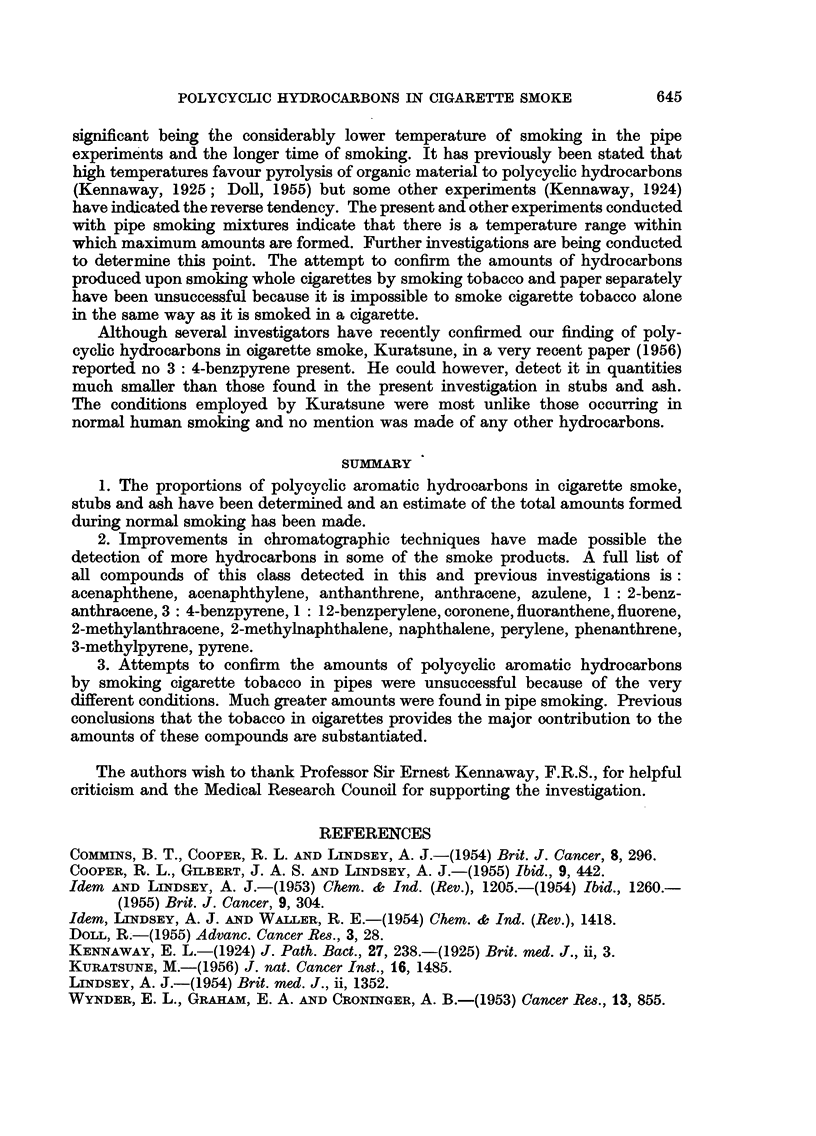# Polycyclic Hydrocarbons in Cigarette Smoke: The Amounts Held in Stubs and Ash

**DOI:** 10.1038/bjc.1956.76

**Published:** 1956-12

**Authors:** J. A. S. Gilbert, A. J. Lindsey


### POLYCYCLIC HYDROCARBONS IN CIGARETTE SMOKE: THE AMOUNTS HELD IN STUBS AND ASH

J. A. S. GILBERT AND A. J. LINDSEY

*From the Department of Chemistry, Sir John Cass College, London, E.C.3*

Received for publication August 16, 1956

IN earlier publications (Cooper, Lindsey and Waller, 1954; Cooper and Lindsey, 1953, 1954, 1955), the amounts of a number of polycyclic hydrocarbons present in mainstream cigarette smoke produced by a method resembling human smoking were determined and, by smoking "cigarettes" prepared entirely from paper, an estimate was made of the amounts produced by the paper of a normal cigarette (Cooper, Gilbert and Lindsey, 1955). The present investigation is an attempt to determine the total amounts of these compounds produced by finding the amounts retained in the stubs and ash of cigarettes, and proves that cigarette tobacco smoked in a pipe gives rise to the same hydrocarbons although the conditions of smoking are different.

### EXPERIMENTAL

The glass apparatus was cleaned and all solvents and reagents were purified as described previosly (Commins, Cooper and Lindsey, 1954). Smoking experiments were carried out with the machine formerly used and the conditions of smoking were as then described.

### *Examination of stubs*

Stubs, of average length 1·5 cm., dried *in vacuo* over sulphuric acid, were extracted to exhaustion with cyclohexane in a Soxhlet extractor and the resulting solution washed three times in turn with 2N sulphuric acid, water, 2N sodium hydroxide, and water. The hydrocarbons were then determined in the neutral solution by the method previously described. The quantities of the various hydrocarbons from 500 cigarette stubs are listed in Table I.

TABLE I.—*Polycyclic Hydrocarbons in Stubs from 500 Cigarettes in Micrograms*

| | |
|---|---|
| Acenaphthene . . . . | 31·5 |
| Acenaphthylene . . . | 18·8 |
| Fluorene . . . . | 39·6 |
| Phenanthrene . . . | 37·5 |
| Anthracene . . . . | 58·3 |
| Pyrene . . . . | 26·7 |
| Fluoranthene . . . . | 181·0 |
| 1 : 2-Benzanthracene . . | 42·1 |
| Perylene . . . . | 1·6 |
| 3 : 4-Benzpyrene . . . | 9.8 |
| Anthanthrene . . . | 1·4 |
| Coronene . . . . | 54·8 |

Naphthalene and 2-methylnaphthalene were also detected.

*Examination of ash*

The ash from cigarettes smoked mechanically in the standardized manner was collected, dried *in vacuo* over sulphuric acid and extracted to exhaustion in a Soxhlet extractor with cyclohexane. The extract was analysed in the manner previously described and the quantities found are tabulated in Table II. Approximately 38 g. of ash are produced from 500 cigarettes and the amounts are computed for this figure.

TABLE II.—*Polycyclic Hydrocarbons in Ash from* 500 *Cigarettes in Micrograms*

| | |
|---|---|
| Acenaphthene | 3·9 |
| Acenaphthylene | 0·8 |
| Fluorene | 1·7 |
| Phenanthrene | 0·08 |
| Anthracene | 0·8 |
| Pyrene | 0·5 |
| Fluoranthene | 0·4 |
| 1 : 2-Benzanthracene | 0·5 |
| 3 : 4-Benzpyrene | 0·3 |
| Coronene | 0·1 |

Azulene and 2-methylnaphthalene were also detected.

*Examination of cigarette tobacco*

The tobacco was obtained by opening cigarettes of the same type as were used in the previous experiments ; it was then smoked in a pipe of pyrex glass attached to the smoking machine. The conditions of smoking were made to conform more closely to those of pipe smoking than to those of cigarette smoking ; that is by using more frequent and shorter puffs until only a small " dottle " of tobacco remained. The total smoking time was considerably longer than was necessary with the same weight of cigarettes. The smoke was collected in cyclohexane flasks and the long glass precipitator previously employed and the analysis carried out exactly as described for cigarette smoke. The quantities of various hydrocarbons from 440 g. of tobacco are listed in the first column of Table III (this quantity was chosen because it is the amount consumed when 500 cigarettes are smoked in the standardized manner).

TABLE III.—*Polycyclic Hydrocarbons from Cigarette Tobacco and Paper*

(Amounts in Micrograms)

| | From 440 g. tobacco | From 16·7 g. paper | Total |
|---|---|---|---|
| Acenaphthylene | 132·0 | 1·4 | 133·4 |
| Anthracene | 104·0 | 0·7 | 104·7 |
| Pyrene | 200·2 | 2·9 | 203·1 |
| 3 : 4-Benzpyrene | 34·9 | 0·7 | 35·6 |

*Temperature measurements*

Average temperatures in the smouldering " coal " in various smoking experiments have previously been reported (Wynder, Graham and Croninger, 1953 ; Lindsey, 1954 ; Doll, 1955). The latest measurements were made with a thermocouple of gold-palladium and platinum-iridium alloys (" Pallador " thermojunction) inserted into the smoking material. The electromotive force produced was measured by means of a precision potentiometer with the cold junction in melting ice, and the calibration was confirmed by standardizing with pure molten zinc. Surface temperatures were measured with a disappearing filament pyrometer. The results are tabulated below :—

| | Quiescent combustion temperature (° C.) | Suction combustion temperature (° C.) | Surface temperature (° C.) |
|---|---|---|---|
| Ordinary cigarette | 650 | 700 | 900+ |
| Paper cigarette | Varies | 655 | 900+ |
| Pipe | ,, | 470 | 700+ |
| Cigar (Havana) | 400–500 | 560 | 800+ |

### DISCUSSION

The examination of stubs and ash gives information that, with the figures previously reported (Cooper, Gilbert and Lindsey, 1955), provides additional knowledge on the distribution of the combustion products during normal smoking. Thus in Table IV are listed the hydrocarbons found in the smoke from 500 cigarettes and, for comparison, the amounts condensed in the stubs and ash. Four important hydrocarbons are thus studied ; those for which determinations have been repeatedly made in smoke of various kinds.

TABLE IV.—*Polycyclic Hydrocarbons found on Smoking* 500 *Cigarettes*

(Amounts in Micrograms)

| | Mainstream smoke | Stubs | Ash | Total |
|---|---|---|---|---|
| Acenaphthylene | 20·5 | 18·9 | 0·8 | 40·2 |
| Anthracene | 48·0 | 59·0 | 0·8 | 107·8 |
| Pyrene | 55·0 | 26·7 | 0·5 | 82·2 |
| 3 : 4-Benzpyrene | 4·0 | 10·0 | 0·3 | 14·3 |

It is noteworthy that, of the total hydrocarbon amounts, a considerable fraction remains in the stubs and further that the proportions of the various hydrocarbons in the stubs are not those found in the mainstream smoke. This could be ascribed to selective absorption in the stubs, but, bearing in mind the physical nature of the smoke—which is an aerosol with the disperse phase consisting of fine viscous droplets—is more probably due to a fractionation process in which the readily aggregated smoke droplets are repeatedly re-volatilized as the smoking proceeds. Thus the material retained in the stubs is richer in the less volatile constituents. It is also likely that, in addition to this redistillation process, there are specific effects due to compounds in the unburnt portion acting as solvents for or combining with the smoke constituents. Simple volatility is certainly not the only effect or more pyrene would be retained in the stubs.

There was no evidence that selective condensation occurred in previous experiments on " Denicotea " filters (Cooper and Lindsey, 1955) where the relative proportions of the hydrocarbons were found to be approximately the same as in the mainstream smoke. Here the temperature remains low and little redistillation would be expected.

The results of the pipe smoking together with those of paper smoking are shown in Table III. In these experiments exactly the same materials were smoked as those described in Table IV but the total amounts of hydrocarbons produced are much greater. The conditions of smoking are very different ; the most significant being the considerably lower temperature of smoking in the pipe experiments and the longer time of smoking. It has previously been stated that high temperatures favour pyrolysis of organic material to polycyclic hydrocarbons (Kennaway, 1925; Doll, 1955) but some other experiments (Kennaway, 1924) have indicated the reverse tendency. The present and other experiments conducted with pipe smoking mixtures indicate that there is a temperature range within which maximum amounts are formed. Further investigations are being conducted to determine this point. The attempt to confirm the amounts of hydrocarbons produced upon smoking whole cigarettes by smoking tobacco and paper separately have been unsuccessful because it is impossible to smoke cigarette tobacco alone in the same way as it is smoked in a cigarette.

Although several investigators have recently confirmed our finding of polycyclic hydrocarbons in cigarette smoke, Kuratsune, in a very recent paper (1956) reported no 3 : 4-benzpyrene present. He could however, detect it in quantities much smaller than those found in the present investigation in stubs and ash. The conditions employed by Kuratsune were most unlike those occurring in normal human smoking and no mention was made of any other hydrocarbons.

### SUMMARY

1. The proportions of polycyclic aromatic hydrocarbons in cigarette smoke, stubs and ash have been determined and an estimate of the total amounts formed during normal smoking has been made.

2. Improvements in chromatographic techniques have made possible the detection of more hydrocarbons in some of the smoke products. A full list of all compounds of this class detected in this and previous investigations is: acenaphthene, acenaphthylene, anthanthrene, anthracene, azulene, 1 : 2-benzanthracene, 3 : 4-benzpyrene, 1 : 12-benzperylene, coronene, fluoranthene, fluorene, 2-methylanthracene, 2-methylnaphthalene, naphthalene, perylene, phenanthrene, 3-methylpyrene, pyrene.

3. Attempts to confirm the amounts of polycyclic aromatic hydrocarbons by smoking cigarette tobacco in pipes were unsuccessful because of the very different conditions. Much greater amounts were found in pipe smoking. Previous conclusions that the tobacco in cigarettes provides the major contribution to the amounts of these compounds are substantiated.

The authors wish to thank Professor Sir Ernest Kennaway, F.R.S., for helpful criticism and the Medical Research Council for supporting the investigation.

### REFERENCES

COMMINS, B. T., COOPER, R. L. AND LINDSEY, A. J.—(1954) *Brit. J. Cancer*, **8**, 296.

COOPER, R. L., GILBERT, J. A. S. AND LINDSEY, A. J.—(1955) *Ibid.*, **9**, 442.

*Idem* AND LINDSEY, A. J.—(1953) *Chem. & Ind.* (*Rev.*), 1205.—(1954) *Ibid.*, 1260.—(1955) *Brit. J. Cancer*, **9**, 304.

*Idem*, LINDSEY, A. J. AND WALLER, R. E.—(1954) *Chem. & Ind.* (*Rev.*), 1418.

DOLL, R.—(1955) *Advanc. Cancer Res.*, **3**, 28.

KENNAWAY, E. L.—(1924) *J. Path. Bact.*, **27**, 238.—(1925) *Brit. med. J.*, ii, 3.

KURATSUNE, M.—(1956) *J. nat. Cancer Inst.*, **16**, 1485.

LINDSEY, A. J.—(1954) *Brit. med. J.*, ii, 1352.

WYNDER, E. L., GRAHAM, E. A. AND CRONINGER, A. B.—(1953) *Cancer Res.*, **13**, 855.